# Comparative Genomics of Clinical and Environmental Isolates of *Vibrio* spp. of Colombia: Implications of Traits Associated with Virulence and Resistance

**DOI:** 10.3390/pathogens10121605

**Published:** 2021-12-10

**Authors:** Alejandra Pérez-Duque, Andrea Gonzalez-Muñoz, Jorge Arboleda-Valencia, Lizbeth Janet Vivas-Aguas, Tania Córdoba-Meza, Ghennie Tatiana Rodriguez-Rey, Paula Díaz-Guevara, Jaime Martinez-Urtaza, Magdalena Wiesner-Reyes

**Affiliations:** 1 Centro de Bioinformática y Biología Computacional BIOS, Manizales 170002, Colombia; alejandra.mapd@gmail.com (A.P.-D.); andrea.gonzalez@bios.co (A.G.-M.); 2 BIONAT: Grupo de Investigaciones en Biodiversidad y Recursos Naturales, Universidad de Caldas, Manizales 170002, Colombia; ghennie.rodriguez@ucaldas.edu.co; 3 Grupo FITOBIOL, Instituto de Biología, Facultad de Ciencias Exactas y Naturales, Universidad de Antioquia, Medellín 050010, Colombia; 4 Programa Calidad Ambiental Marina, Instituto de Investigaciones Marinas y Costeras José Benito Vives De Andreis INVEMAR, Santa Marta 470006, Colombia; janet.vivas@invemar.org.co (L.J.V.-A.); tania.cordoba@invemar.org.co (T.C.-M.); 5 Grupo de Microbiología, Instituto Nacional de Salud, Bogotá 111321, Colombia; pdiaz@ins.gov.co; 6 Department of Genetics and Microbiology, Faculty of Biosciences, Universitat Autonoma de Barcelona, 08193 Bellaterra, Spain; jaime.martinez.urtaza@uab.cat

**Keywords:** vibriosis, virulence, antibiotic resistance, pangenome, whole genome sequencing

## Abstract

There is widespread concern about the increase in cases of human and animal infections caused by pathogenic *Vibrio* species due to the emergence of epidemic lineages. In Colombia, active surveillance by the National Institute of Health (INS) has confirmed the presence of *Vibrio*; however, in routine surveillance, these isolates are not genomically characterized. This study focused on the pangenome analysis of six *Vibrio* species: *V. parahaemolyticus*, *V. vulnificus*, *V. alginolyticus*, *V. fluvialis*, *V. diabolicus* and *V. furnissii* to determine the genetic architectures of potentially virulent and antimicrobial resistance traits. Isolates from environmental and clinical samples were genome sequenced, assembled and annotated. The most important species in public health were further characterized by multilocus sequence typing and phylogenomics. For *V. parahaemolyticus*, we found the virulent ST3 and ST120 genotypes. For *V. vulnificus*, we identified isolates belonging to lineages 1 and 2. Virulence gene homologues between species were found even in non-pathogenic species such as *V. diabolicus*. Annotations related to the mobilome, integrative mobile and conjugative elements and resistance genes were obtained from environmental and clinical isolates. This study contributes genomic information to the intensified surveillance program implemented by the INS to establish potential sources of vibriosis in Colombia.

## 1. Introduction

The genus *Vibrio* comprises bacterial species present as free-living organisms in aquatic and marine habitats, or associated with fish and marine invertebrates. Several species of this bacterial genus cause food-borne diseases and wound infections resulting from contact with water or raw seafood. The number of cases of human infection by *Vibrio* species have increased worldwide. The diseases caused by *Vibrio* spp. are divided into two large groups: cholera, caused by enteropathogenic *Vibrio cholerae* and vibriosis, caused by other pathogenic *Vibrio* species [1]. Cholerae is a rapidly disseminating diarrheal disease that causes quick dehydration and can be fatal if not treated on-time [2]. Patients with vibriosis present a variety of symptoms depending on the infecting *Vibrio* species, infection route and host susceptibility. Symptoms range from gastrointestinal illness, such as gastroenteritis, to extraintestinal pathologies, including skin infections and septicemia [1].

*Vibrio* is commonly found in the United States of America, where the number of cases of infection has increased since 2000, reaching approximately 80,000 cases and 100 deaths annually [3]. Similarly, countries in Asia reported impacts of *Vibrio* infections on humans and massive death of marine animals, affecting the seafood industry [4]. In Europe, the presence of *Vibrio vulnificus, Vibrio parahaemolyticus* and other *Vibrio* species has raised concerns regarding their potential to generate significant clinical problems and outbreaks associated with climate change [5]. In America, alerts have been raised throughout the continent due to the cholera outbreaks in Haiti and the Dominican Republic in 2010 and 2011, as well as case reports in Cuba, Venezuela and Mexico [6].

Colombia is not exempt from possible cholera or vibriosis outbreaks due to its geographic location and the presence of the Atlantic and Pacific coasts [7]. Although *V. cholerae*, *V. parahaemolyticus* and *V. vulnificus* are the main reported pathogens, there is also concern regarding an increase in cases of infection by other *Vibrio* species and the emergence of epidemic lineages [8]. A vibriosis outbreak would affect the economic sector of the Atlantic and Pacific coasts of Colombia, especially, seafood production and tourism.

In response to this contingency, the Instituto Nacional de Salud (National Institute of Health, INS) of Colombia reinforced the Intensified Cholera Surveillance program in 2010 [9]. Although Colombia is currently in an inter-epidemic period, the intensified surveillance between 2010 and 2015 recovered 502 probable *V*. *cholerae* isolates (e.g., toxin negative and positive), as well as other *Vibrio* species. The presence of these isolates suggests that the country is at risk for a possible reemergence of cholera and vibriosis. Furthermore, the recovered isolates belonging to non-cholera vibrios have not been genotyped or characterized at the genome level, so it is unknown if these isolates carry toxin or virulence genes and antimicrobial resistance traits [10]. Therefore, genetic characterization studies are important to understand the public health risks associated with the presence of these bacteria in the environment [11,12,13,14,15]. Additionally, continuous monitoring of the genotypes is essential to determine their pathogenicity and to establish potential sources of epidemic outbreaks. This information is relevant for adopting timely measures in response to a contingency, allowing to reduce the impact of this pathogen on the country.

Species of *Vibrio* have highly plastic genomes since there is a high probability of horizontal transfer of virulence and antibiotic resistance genes from virulent to non-virulent strains due to their free-living nature. Whole genome sequencing of several *Vibrio* isolates and comparative genomics analyses have revealed a wide array of mutations, chromosomal rearrangements and gene gain and loss events resulting from duplication or horizontal gene transfer [16]. Virulence genes are normally identified within mobile genetic elements; therefore, these elements can favor the appearance of novel virulent strains [17]. The *Vibrio* genome comprises a core genome composed of conserved genes necessary for essential functions and a flexible gene pool, including mobile elements, virulence factors and antibiotic resistance determinants, among others, that are involved in adaptation processes [18]. Pangenome analyses represent an adequate comparative genomics approach to study the intraspecific diversity of *Vibrio* by analyzing the core and accessory genomes of different isolates of a species.

This research involved species-level pangenome analyses to genetically characterize isolates of six non-cholera *Vibrio* species that were obtained from environmental and clinical samples in Colombia to identify potential sources of vibriosis. Furthermore, this study relied on whole genome sequencing (WGS) as a high-throughput and high-resolution approach to characterize isolates for genomic surveillance in epidemiology. The pangenome allowed us to contrast the diversity within the *Vibrio* species analyzed and the virulence and antibiotic-resistance determination allow us to identify the potential pathogenesis of the isolates. For the most important species in public health, the multilocus sequence typing (MLST) approach was used to determine pandemic clones of *V*. *parahaemolyticus* and phylogenetic analyses allowed effectively identifying lineages of *V*. *vulnificus*. Coupled with isolate source information (e.g., environmental or clinical sample, geographic location), this study contributes to identifying potential environmental sources of vibriosis. This is relevant for the intensified cholera surveillance program implemented by the National Institute of Health of Colombia that aims to predict future vibriosis outbreaks in the country.

## 2. Results

### 2.1. Characteristics of the Assembled Genomes of Vibrio spp.

**Figure 1:**
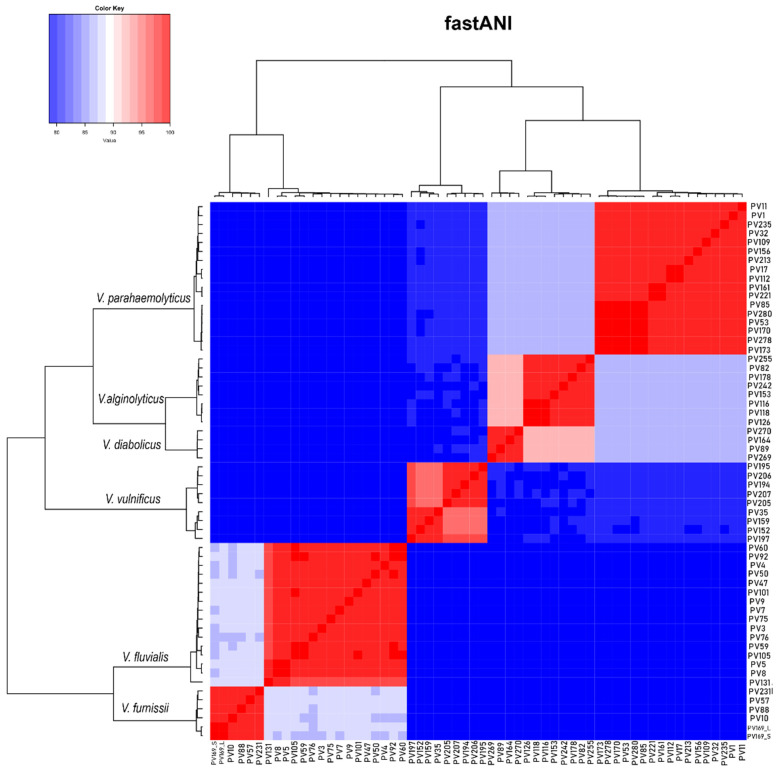
Heatmap based on hierarchical clustering of the Whole-genome Average Nucleotide Identity (ANI) calculated for the 60 *Vibrio* spp. isolates of the study.

A total of 60 isolates were analyzed in this study, belonging to *V. parahaemolyticus* (n = 17)*, V. vulnificus* (n = 9)*, V. alginolyticus* (n = 8), *V. fluvialis* (n = 16)*, V. diabolicus* (n = 4) and *V. furnissii* (n = 6). General characteristics of the obtained assemblies, such as assembled length, number of scaffolds, %GC, N50 length, number of coding sequences (CDS) and reference genome fraction are reported in Supplementary Table S1. The assembled genomes showed a similar size and GC content to the corresponding reference genome of each species. Within each species, the strains shared more than 95% average nucleotide identity (ANI). Moreover, among species, the most genetically similar were *V. alginolyticus* and *V. diabolicus* with ~90% ANI and *V. fluvialis* and *V. furnissii* with ~85% ANI (Figure 1).

### 2.2. Vibrio parahaemolyticus

Of the 17 *V. parahaemolyticus* isolates (seven clinical, ten environmental), we found seven environmental isolates with new sequence types, six clinical isolates assigned to ST3, one clinical isolate assigned to ST120 and two untypeable isolates (Supplementary Table S4).

**Figure 2:**
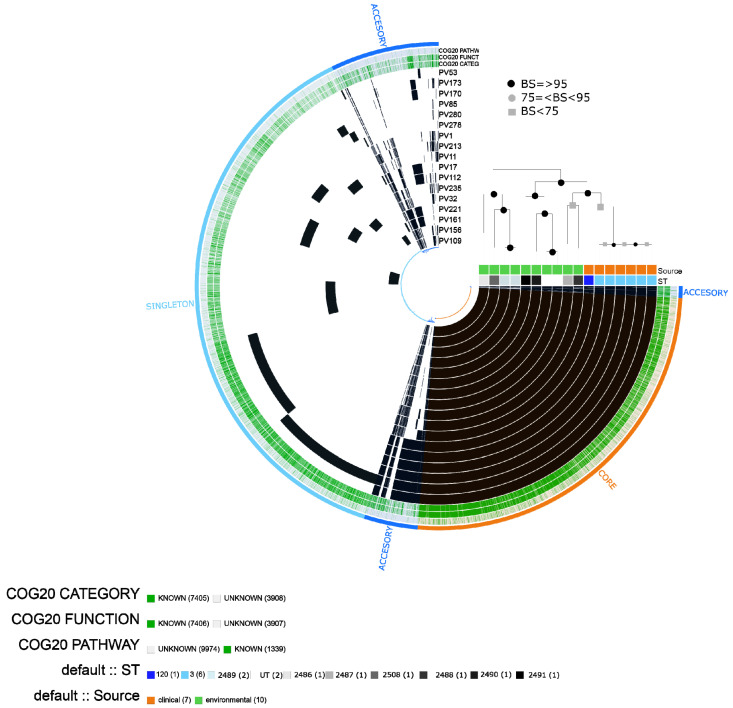
*Vibrio parahaemolyticus* pangenome based on 17 isolates analyzed. Presence/absence of gene clusters are represented by black and gray bars, respectively. Each bin within the pangenome is signaling core, accessory and singleton fractions. The labels represent in order: Number of gene clusters assigned to COG20_CATEGORY, COG20_FUNCTION and COG20_PATHWAY; each isolate (PV); information on ST; and source of the isolate (clinical/environmental). The pangenome is ordered according to a maximum likelihood (ML) tree based on 2149 single-copy concatenated core genes. Bootstrap supports (BS) are based on 100 replicates.

The pangenome of *V. parahaemolyticus* comprised a total of 11313 gene clusters, including 7405 that were assigned to COG (cluster of orthologous of proteins). The core genome contained 3859 gene clusters, the accessory genome 1747 and the singletons 5707. In the pangenome scheme, we observed a defined block of gene clusters belonging to ST3 and a block of gene clusters shared by clinical isolates (ST120 and ST3). The isolates belonging to ST3 were grouped together in the maximum likelihood (ML) tree (Figure 2).

In the clinical group, we found a significant enrichment of categories (adjusted q-value ≤ 1.03E-02) associated with: [N] cell motility, [U] intracellular trafficking, secretion and vesicular transport related to type III secretory pathway and type VI secretion system, including proteins related to flagella and other component proteins. In contrast, the most enriched categories in the environmental isolates were associated with cell cycle control, cell division, carbohydrate, amino acid and nucleotide transport and metabolism (Table 1).

The *tlh* (thermolabile hemolysin) gene, MAM7 (multivalent adhesion molecule) and genes related to T3SS1 were found in 100% of the *V. parahaemolyticus* isolates (n=17). In support of the pangenome results, a cluster of virulence genes was observed in all ST3 (n = 6) and ST120 (n = 1) clinical isolates, whereas it was absent in the environmental isolates. This cluster included *tdh* (thermostable direct hemolysin A) and different proteins belonging to the type III secretion system (T3SS1 and T3SS2), including effector proteins (*vopC, vopT, vopZ*, VPA1331, *vopA/vopP, vopL*), translocator proteins (*vopD2, vopB2*), transcriptional regulator proteins (*vtrA*), C-ring proteins (*vscT2, vscR2*), chaperones (VPA1363) and other proteins and putative proteins (*VscS2, VscC2, VscQ2, vscU2, vcrD2, vscJ2*, VPA1337, *vscN2)*.

**Figure 3:**
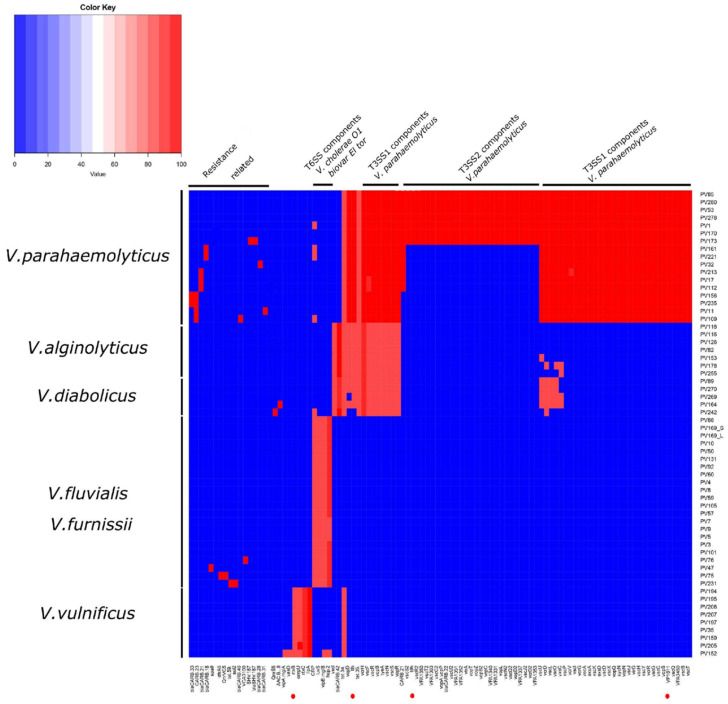
Heatmap of the in silico predicted virulence and resistance genes of the 60 *Vibrio* spp. isolates grouped according to sequence identity. This analysis allowed the separation between species and between clinical and environmental *V. parahaemolyticus* isolates due the different virulence profile predicted for each, except for *V. fluvialis* and *V. furnissii*.

Regarding antibiotic resistance, the most predicted genes were related to the tetracycline efflux pump *tet(35)* and resistance to beta-lactamases (CARB-21, CARB-23), which were found in the 17 isolates of *V. parahaemolyticus*. Four isolates (including three environmental) contained the cAMP receptor protein (CRP), which is a global regulator that represses MdtEF multidrug efflux pump expression associated with resistance to fluoroquinolone, macrolide and penam [19]. One clinical isolate (e.g., PV173) presented an additional gene (SHV-187) related to resistance to carbapenem, cephalosporin and penam, according to CARD (Figure 3).

In isolate PV173 (ST3), we found a putative integrative and mobilizable element (IME) with a size of ~80kb containing virulence genes related to T3SS2 and other genes such as *acfD*, *tdh*, *flhA*. In PV1 (ST120), we found a putative integrative and conjugative element (ICE) with T4SS containing virulence genes related to MSHA type IV pilus (Table 2).

### 2.3. Vibrio vulnificus

Of the nine *V. vulnificus* isolates (eight environmental and one clinical), one isolate (PV207) could be assigned to a pre-existing MLST profile (ST335), while the other eight isolates were assigned to new sequence types (Supplementary Table S5).

We obtained a total of 6806 gene clusters, including 4504 assigned to COG functions. The pangenome contained 3510 core genes, 1069 accessory genes and 2224 singletons. Due to the low number of clinical isolates (n = 1), the differential analysis of enrichment categories was not done. Instead, we observed that all isolates contained categories related to important pathogenicity traits, such as [P] Inorganic ion transport and metabolism related to iron uptake, [U] Vesicular intracellular traffic, secretion and transport, [O] Post-translational modification, protein turnover, chaperones related to metalloproteases; [Q] Secondary metabolites biosynthesis, transport and catabolism, [N] Cell motility related to flagella, [T] Signal transduction mechanisms related to chemotaxis, among others.

**Figure 4:**
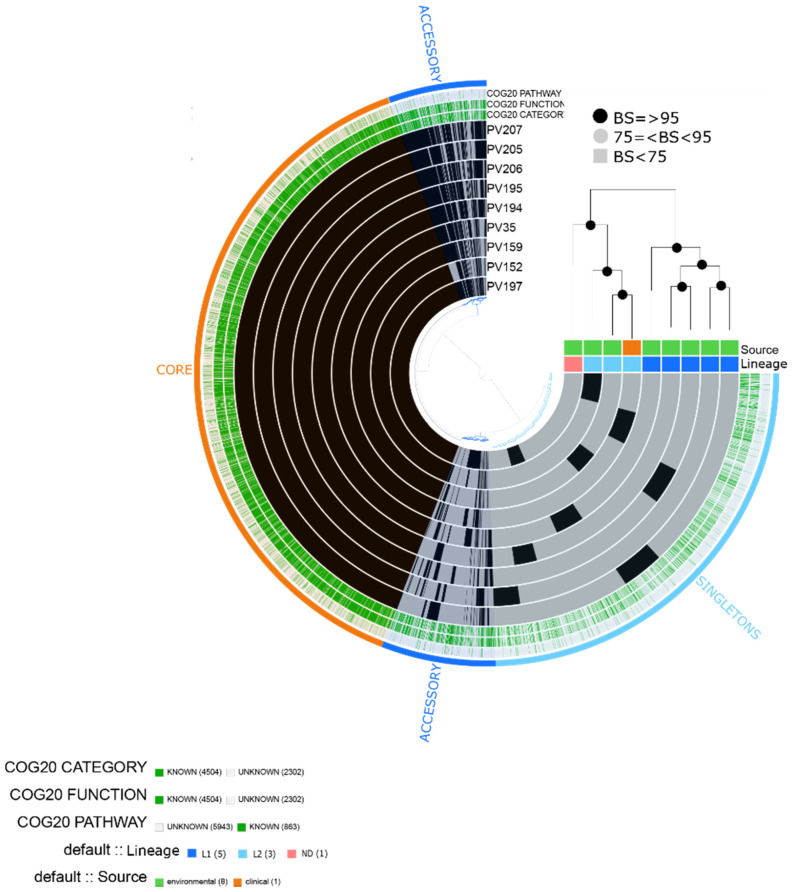
*Vibrio vulnificus* pangenome. The labels represent in order: number of gene clusters assigned to COG20_CATEGORY, COG20_FUNCTION and COG20_PATHWAY, lineage (L1 or L2) and source (clinical or environmental). Maximum likelihood tree based on 2429 single-copy concatenated core genes. Bootstrap supports (BS) are based on 100 replicates.

Based on the core genome phylogeny, the nine isolates were grouped into three clusters (Figure 4). Furthermore, the phylogenetic analysis (Supplementary Figure S1) and the whole-genome average nucleotide identity (Supplementary Figure S2) based on alignments to 80 reference *V. vulnificus* isolates obtained from [20], showed that five environmental isolates belonged to Lineage 1 and two environmental isolates and one clinical to Lineage 2. In the whole genome average nucleotide identity analyses, isolate PV197 showed high similarity to the reference isolate V252, assigned to lineage 5. However, despite this result, these two isolates did not form a monophyletic group in the phylogenetic analyses.

In 100% of the *V. vulnificus* isolates, we found virulence genes homologous to *V. cholerae* O1 biovar El Tor, including genes related to RTX toxin activation (*rtxC*) and transportion (*rtxB*). Additionally, *ompU* and *IlpA* were found in all isolates. Regarding resistance, we predicted a gene related to tetracycline resistance *tet*(34) in all the isolates (Figure 3).

### 2.4. Vibrio alginolyticus

**Figure 5:**
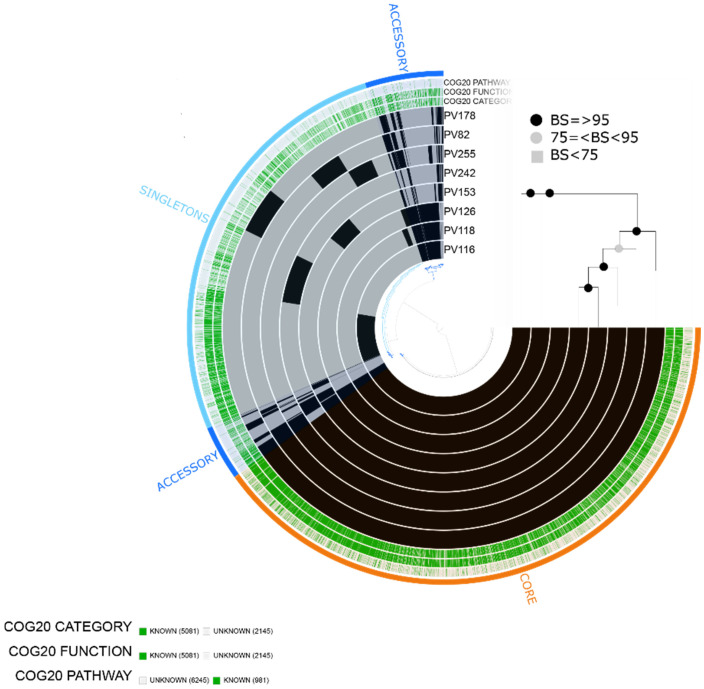
*Vibrio alginolyticus* pangenome. The labels represent in order: number of gene clusters assigned to COG20_CATEGORY, COG_20FUNCTION and COG_20 PATHWAY. Maximum likelihood tree based on 2853 single-copy concatenated core genes. Bootstrap supports (BS) are based on 100 replicates.

For the eight environmental isolates of *V. alginolyticus*, we obtained a total of 7226 gene clusters, including 5081 assigned to COG functions. The pangenome contained 3866 core genes, 808 accessory genes and 2550 singletons. Categories related to virulence were found in the eight isolates, namely [N|W] Cell motility |Extracellular structures related to type IV pilus and type II secretory pathway, [O] Posttranslational modification, protein turnover, chaperones related to proteases, [T] Signal transduction mechanisms related to chemotaxis, [V] Defense mechanisms, [R] General function prediction only related to putative hemolysin, [P] Inorganic ion transport and metabolism related to iron utilization and transport, among others. Based on the phylogenetic analyses, we observed a group composed of PV116, PV118 and PV126, which shared a specific cluster of genes mainly related to [Q] Secondary metabolites biosynthesis, transport and catabolism [T] Signal transduction mechanisms [P] Inorganic ion transport and metabolism, [P] coenzyme transport and metabolism, [C] Energy production and conversion, among others, that could be involved in adaptation to specific hosts or environments (Figure 5).

The eight isolates contained the *tlh* gene and genes of the T3SS1 secretion system, such as *VopD*, *VopB*, *VcrH*, *tyeA*, *vscN*, *vscR*, *vscS*, *vopR*, *vscI* and *vscF*. Similar to *V. parahaemolyticus*, the eight isolates of *V. alginolyticus* shared the predicted gene *tet(35)* related to the tetracycline efflux pump. One isolate (PV242) presented CRP and a plasmid-mediated quinolone resistance protein (QnrS5) (Figure 3).

### 2.5. Vibrio diabolicus

**Figure 6:**
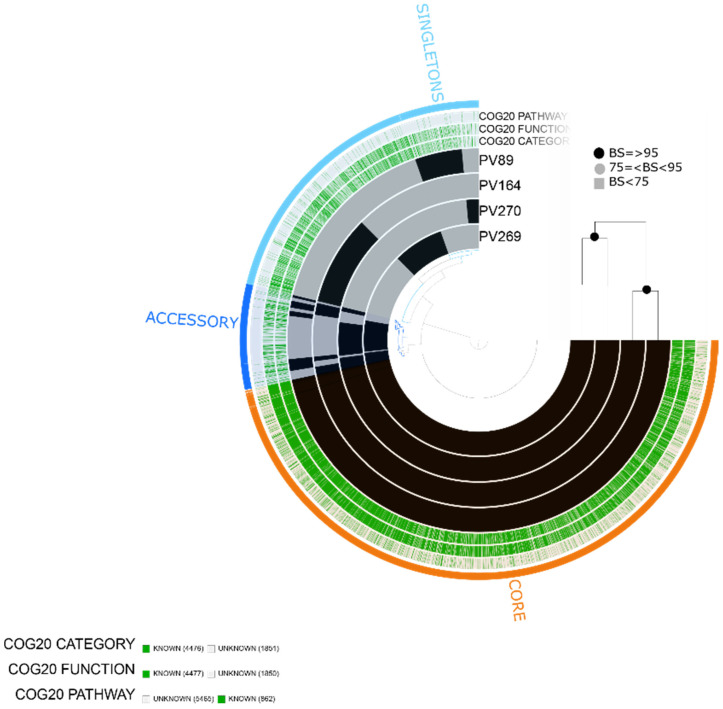
*Vibrio diabolicus* pangenome. The labels represent in order: number of clusters of genes assigned to COG20_CATEGORY, COG20_FUNCTION and COG20_PATHWAY. Maximum likelihood tree based on 2780 single-copy concatenated core genes. Bootstrap supports (BS) are based on 100 replicates.

We predicted 6327 total genes, including 4476 assigned to COG functions. There were 3922 core genes, 599 accessory genes and 1788 singleton genes in the pangenome based on the four *V. diabolicus* environmental isolates. Similar to *V. alginolyticus*, we found the following functional categories related to virulence: [N|W] Cell motility |Extracellular structures related to type IV pilus and flagella-related proteins, [U] Intracellular trafficking, secretion and vesicular transport related to type II secretion pathway, [Q] Secondary metabolites biosynthesis, [T] Signal transduction mechanisms related to chemotaxis, [V] Defense mechanisms, [P] Inorganic ion transport and metabolism related to iron utilization, transport and siderophore, among others. The isolates were grouped into two clusters comprising isolates PV270/PV269 and PV89/PV164. Furthermore, PV270 and PV269 shared a gene block with functional categories mainly related to [G] Carbohydrate transport and metabolism, [D] Cell cycle, cell division, chromosome partitioning, [C] Energy production and conversion, among others (Figure 6).

We found that the virulence profile of *V. diabolicus* was similar to that of *V. alginolyticus* since both species shared genes related to T3SS1. A cluster of virulence genes was found with additional genes related to T3SS1 (*vscU*, *vcrD, vscL*, *vscO, YscO* and *vxsC*). Similar to *V. parahaemolyticus* and *V. alginolyticus*, the four *V. diabolicus* isolates contained the *tet(35)* antibiotic resistance determinant gene (Figure 3).

### 2.6. Vibrio fluvialis

**Figure 7:**
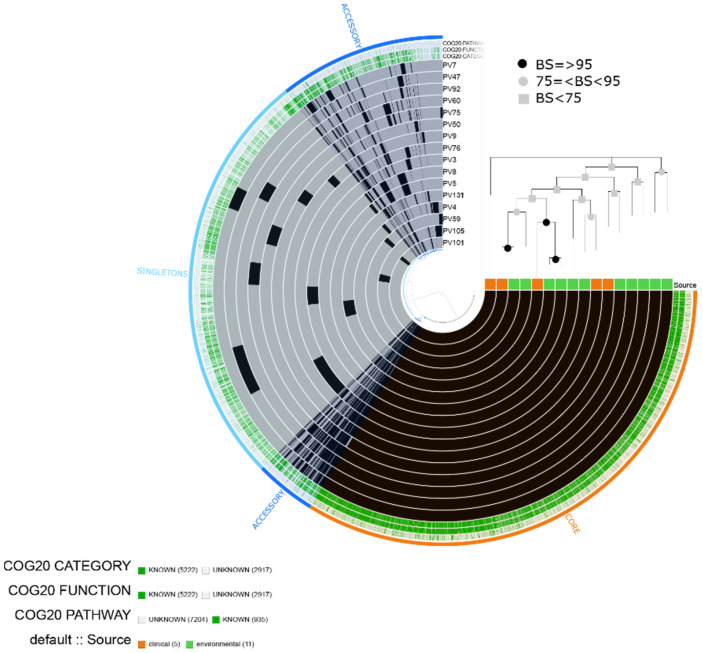
*Vibrio fluvialis* pangenome. The labels represent in order: number of clusters of genes assigned to COG20 CATEGORY, COG20_FUNCTION and COG_20 PATHWAY and isolate source (clinical or environmental). Maximum likelihood tree based on 2382 single-copy concatenated core genes. Bootstrap supports (BS) are based on 100 replicates.

We predicted a total of 8139 gene clusters (5222 assigned to COG functions) distributed into 3666 core genes, 1574 accessory genes and 2898 singletons, which comprised the pangenome of the 16 isolates of *V. fluvialis* (five clinical isolates and 11 environmental isolates) (Figure 7).

The enrichment test did not show significantly enriched functional categories among the clinical and environmental isolates. Moreover, the phylogenetic analyses did not indicate a differential aggrupation among the isolates and we did not observe specific blocks of gene clusters related to groups within the pangenome. Therefore, we report the categories related to virulence mechanisms in the core genome. We found categories such as [N|U|W] Cell motility|Intracellular trafficking, secretion and vesicular transport|Extracellular structures related to secretory pathway type VI, [R] General prediction only related to putative hemolysins, [Q] Inorganic ion transport and metabolism related to mechanisms implicated in iron uptake, [T] Signal transduction mechanisms related to chemotaxis, among others.

The sequence homology-based search showed that the virulence factors predicted in *V. fluvialis* were mostly associated with *V*. *cholerae* O1 biovar El Tor. In both clinical and environmental isolates, we found genes associated with secretion system type VI (T6SS) including type VI secretion system substrate (*hcp-2*), type VI secretion system tubule-forming protein VipB (*vipB/mglB)* and the *luxS* gene. The 16 isolates contained CRP and one isolate (PV75) presented a predicted gene (*QnrVCS*) related to resistance to quinolone and *dfrA6* related to resistance to trimethoprim (Figure 3).

Out of the 16 isolates, we found predicted IMEs and ICEs in 10 isolates. These elements contained predicted virulence genes such as *cqsA*, *tcpl*, *IlpA*, *motB*, *motA* and *flgD*. Furthermore, in PV60, a predicted ICE contained genes related to flagella (*flaC,flaB,flgA,flgJ*) and chemotaxis (*cheR,cheV*) (Table 2).

### 2.7. Vibrio furnissii

**Figure 8:**
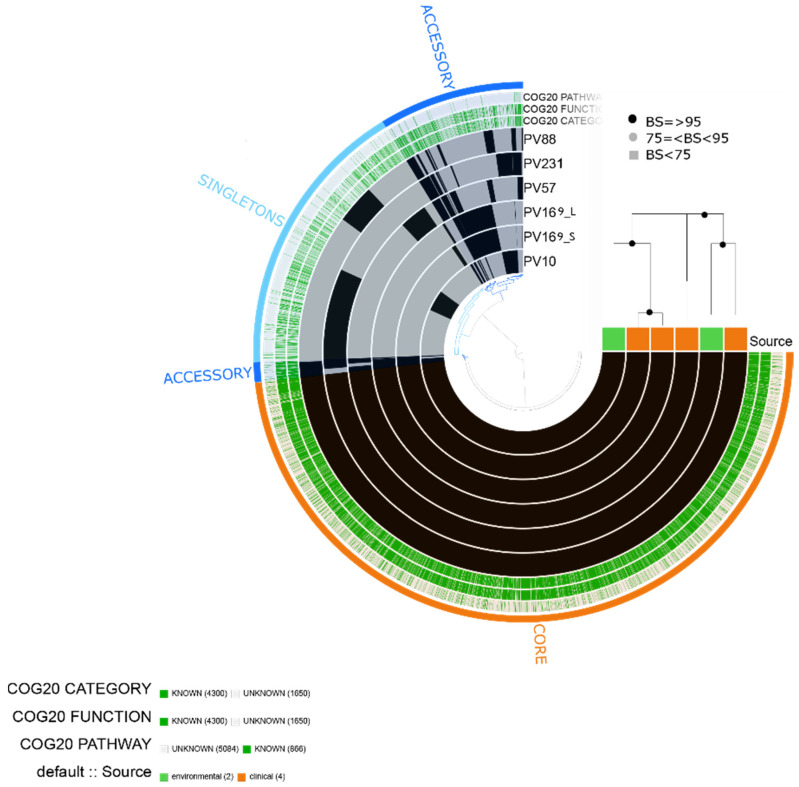
*Vibrio furnissii* pangenome. The labels represent in order: number of clusters of genes assigned to COG20 CATEGORY, COG20_FUNCTION and COG_20 PATHWAY and isolate source (clinical or environmental). Maximum likelihood tree based on 3411 single-copy concatenated core genes. Bootstrap supports (BS) are based on 100 replicates.

We obtained 5950 total genes, including 4300 assigned to COG functions. The pangenome contained 3822 core genes, 787 accessory genes and 1339 singletons based on six *V. furnissii* isolates. The isolates (four clinical and two environmental) did not show significantly enriched categories or differential aggrupation among clinical and environmental isolates. Similar to the results for *V. fluvialis*, the isolates contained gene clusters assigned to virulence-related categories such as [U] Intracellular trafficking, secretion and vesicular transport implicated in diverse components of type VI secretion system; [N] Cell motility related to flagellar biosynthesis proteins [P] Inorganic ion transport and metabolism including mechanisms related to iron uptake such as transport systems, enterobactin and siderophore related [G] Carbohydrate transport and metabolism related to chitinase, among others (Figure 8).

Due to the high similarity between *V. fluvialis* and *V. furnissii*, the isolates also presented a similar virulence profile. Both clinical and environmental isolates of *V. furnissii* contained genes related to the secretion system type VI (T6SS) (*hcp-2*, *vipB/mglB*) and the *luxS* gene. All the isolates contained a predicted CRP. Finally, isolate PV231 contained a predicted *sul2*, which is a resistance determinant associated with plasmids (Figure 3).

## 3. Discussion

This research sets a precedent in genomic surveillance of environmental and clinical isolates of *Vibrio* spp. in Colombia. We characterized the genetic potential for virulence, resistance and the presence of mobile elements in non-cholera vibrio isolates based on WGS and pangenome analyses.

In *Vibrio*, due to the high genomic plasticity, the differentiation of sister species is difficult using traditional phenotypic and biochemical methods for identification. Therefore, closely related species may be misclassified due to the similar phenotypic characteristics [21]. The calculation of average nucleotide identity (ANI) allows classifying prokaryotic species based on a similarity threshold of 95–96%. Our results showed that the isolates from each species shared ~95% ANI. Furthermore, we were able to differentiate between closely related species, such as *V. alginolyticus*/*V. diabolicus* and *V. fluvialis*/*V. furnissii*, based on the genome sequence.

We found the ST3 genotype in six isolates of *V. parahaemolyticus*. This genotype has been associated with serotype O3:K6. This pandemic clone was detected for the first time in Asia and spread through countries in America and Europe. Since 1996, it has been detected in Peru causing outbreaks mainly associated with climate change [22]. In [23], there is a report of a Colombian isolate sampled in 2016 belonging to genotype ST3. The virulence profile of this isolate was *tdh* + and *trh* -, congruent with the laboratory determination by PCR of *trh* and *tdh* of the *V. parahaemolyticus* isolates of the present study, where 100% (n = 17) were negative to *trh* and seven isolates were positive to *tdh* corresponding to ST3 (n = 6) and ST120 (n = 1) (Supplementary Table S2). The other sequence type found in this study corresponded to one isolate belonging to ST120. This ST was reported in 2009, causing an outbreak in Peru due to an introduction of Asian populations of pathogenic *V*. *parahaemolyticus* to the Pacific coasts of South America [24]. Here, we report the first record of ST120 in Colombia.

Regarding the *in silico* predicted virulence factors, according to [25], *tdh* has been reported in more than 90% of clinical isolates but is rarely present in environmental isolates. Therefore, it is considered a reliable virulence marker. Indeed, virulent strains exhibit certain biological characteristics that differentiate them from non-pathogenic environmental strains. This is congruent with our results since we found significant differences in the gene content and function of the accessory genome in clinical vs. environmental strains. In addition, in addition to *tdh*, the clinical strains (n = 7) presented secretion systems T3SS1 and T3SS2; the former causes cytotoxicity and the latter is essential for enterotoxicity associated with pathogenicity in humans [22]. Boyd et al., [26] suggested that T3SS2 may be an integrative element due to the presence of transposases and low content of GC (40%). This pathogenic island was named *Vibrio parahaemolyticus* island-7 (VpaI-7). We found a putative IME of ~80 kb and 39.38% (GC) in one of the isolated genotyped as ST3. According to [26], this IME could correspond to VPAI-7 and should be present in the potentially pathogenic strains of *V. parahaemolyticus* determined here due to the presence of the associated virulence genes (T3SS2 and *tdh*).

The isolates of *V. vulnificus* were classified according to the lineages proposed by [20]. Here, we found five environmental isolates belonging to Lineage 1. According to [20], this lineage comprises both clinical and environmental strains implicated in human infections due the consumption of seafood and represent the most dangerous strains related to public health. We found one clinical and two environmental isolates associated with lineage 2. The isolates from lineage 2 have been involved in both human and fish infections. The laboratory determination of *vcgC* and *vcgE* of the *V.vulnificus* isolates of the present study (Supplementary Table S2) showed that five of the nine isolates were positive to *vcgC* and, according to our genomic analyses, these isolates belonged to Lineage 1. Regarding Lineage 2, two of the three isolates were positive to *vcgE* and one to *vcgC*. In terms of the virulence of *V. vulnificus*, the *VvhA* and RTX cytotoxins are the most important factors; specifically, the former shares homology with cytotoxins of *V. cholerae*. Here, we found components of RTX in all the isolates of *V. vulnificus*. This cytotoxin form pores on the host cell membrane and causes cell lysis [27]. Other virulence genes found in all the isolates were *OmpU* and *IlpA*. These genes have been classified as outer membrane proteins important in adherence, immune response and cytotoxicity [28].

Gene homology between the virulence mechanisms of *V. alginolyticus* and *V. parahaemolyticus* has been shown [29]. In fact, the *tdh* and *trh* genes in *V. alginolyticus* have been proposed as important virulence markers [30]. Nevertheless, in this study, these genes were not predicted in any of the isolates. Another homologous system to *V. parahaemolyticus* is T3SS1. We found several components of T3SS1 and *tlh* in the environmental isolates of *V. alginolyticus* and *V. diabolicus*. Regarding this system, cytotoxic activity in fish has been described [31]. To date, there are no reports of *V. diabolicus* causing infection; however, Song et al. [32] reported that one virulent isolate in mice was misidentified as *V. alginolyticus*. In fact, the shared virulence profile with *V. alginolyticus* may imply that *V. diabolicus* could be potentially dangerous to aquatic organisms.

Chibani et al., [33] demonstrated in *V. alginolyticus* isolates that the formation of ecotypes is mainly driven by horizontal gene transfer (HGT) since the authors detected a specific gene-block cluster shared by habitat-specific strains and a closed pangenome among them. We found specific clusters of genes shared among some isolates of *V. alginolyticus*, as well as of *V. parahaemolyticus*. As future work, it would be valuable to further analyze these genomic signatures implicated in a specific lifestyle, host or habitat for epidemiological studies. Furthermore, the use of complete genomes is highly recommended in pangenomic studies.

In *V. fluvialis*, both clinical and environmental strains can express putative virulence factors; for instance, one of the most important is the hemolysine *vfh* [34]. In this study, we found genes homologous to T6SS of *V. cholera* in *V. fluvialis* and *V. furnissii*. This secretion system has been implicated in biofilm formation and cytotoxicity and contributes to bacterial pathogenicity by exerting toxic effects on host cells. In *V. fluvialis*, this secretion system and other extracellular proteases and hemolysins are regulated by quorum sensing [35]. Here, we found the *LuxS* gene in all the isolates.

ICEs occur between bacteria by conjugation, resulting in the transfer of several functions, including acquired antimicrobial resistance and virulence factors [36]. Here, the ICEs and IMEs found in the *V. fluvialis* isolates contributed genes related to flagella and TCP. Nevertheless, the contribution of this kind of elements has been related mainly to AMR resistance. For example, [34] mention that plasmid-borne qnrVC-like genes have been reported for quinolone resistance in *V. fluvialis* strains. This study found *QnrVCS* and *QnrSS* integron and plasmid-mediated quinolone resistance proteins in the environmental *V. fluvialis* isolate PV75 and the *V. alginolyticus* isolate PV242. Cattoir et al. [37] found this type of resistance determinant in *Vibrio splendidus* and compared it with other species of the family Vibrionaceae, concluding that Gram-negative species of aquatic environments may be reservoirs of plasmid-mediated Qnr-like determinants. Other resistance determinants related to plasmids and integrons, respectively, were *sul2* in *V. furnissii* (i.e., predicted in the environmental isolate PV231) and *dfrA6* in *V*. *fluvialis* PV75. Related to these elements, [38] found *V. cholerae* isolates with resistance genes related to trimethoprim and sulfamethoxazole (*dfrA1* and *sul2*) detected in ICE’s.

Loo et al. [39] reviewed the incidence of antibiotic resistance in *Vibrio* spp., relating that recently both environmental and clinical isolates have developed resistance to ampicillin, chloramphenicol, tetracycline, streptomycin, kanamycin, trimethoprim and carbapenem. This is consistent with our findings, which showed *tet(35)* and CARB genes encoding for a tetracycline inactivation enzyme and betalactamases in all the *V. parahaemolyticus* isolates. Furthermore, all the isolates of *V. alginolyticus*, *V. diabolicus* and *V. vulnificus* contained a gene related to tetracycline resistance. Another gene widely found in our isolates was CRP, which is a global regulator of multidrug resistance mainly described in *Escherichia coli* [19] and, according to CARD database, shows sequence variants in the resistomes of several Gram-negative bacterial species.

Due to the recent implementation of WGS in surveillance systems, there are some discrepancies between traditional in vitro and in silico methods related to taxonomical classification and gene determination. Genome-level analyses are sensitive for species determination [40]; however, given that discrepancies between in vitro and in silico determinations of antimicrobial resistance have been reported [41], in vitro validations of the presence of predicted antimicrobial genes are highly recommended.

The acquisition of mobile DNA can mediate the appearance of virulent or more virulent strains, even in formerly non-pathogenic species [42]. Despite the limitation of analyzing incomplete scaffold-level genomes, we attempted to determine the presence of mobile elements in the *Vibrio* isolates. We found functional categories assigned to [X] Mobilome: prophages, transposons including phage-related proteins, transposase, transcriptase and plasmid-related components in the accessory and singleton fractions of the six pangenomes. In addition, we found ICE’s and IME’s and resistance genes related to mobile elements, which support mobile elements as important sources of variation in the genus and that environmental isolates could be reservoirs of virulence and resistance genes.

Knowledge of the risk of vibriosis outbreaks due to the presence of potentially virulent and/or resistant isolates is essential for establishing management and control measures to prepare for a contingency and reduce socio-economic impacts of outbreaks. The implementation of WGS provides high resolution to characterize pathogen isolates for genomic surveillance. The results of this study are important to reinforce the intensified laboratory surveillance that is currently conducted by the Microbiology Group of the National Institute of Health of Colombia

## 4. Materials and Methods

### 4.1. Study Area and Sampling

A total of 60 isolates were analyzed in this study, including *V. parahaemolyticus* (n = 17), *V. vulnificus* (n = 9), *V. alginolyticus* (n = 8), *V. fluvialis* (n = 16), *V. diabolicus* (n = 4) and *V. furnissii* (n = 6). These isolates were recovered from water samples with three different salinity conditions (marine, estuarine and continental/fresh water) and clinical samples (stool and wound samples) (Supplementary Table S2). The isolates were obtained from two different programs in Colombia. One was the surveillance network for the conservation and protection of marine water and coasts of Colombia (*Red de Vigilancia para la conservación y Protección de las Aguas Marinas y Costeras de Colombia*-REDCAM- http://www.invemar.org.co/redcam accessed on 2 August 2021), which evaluated 38 monitoring stations distributed in the departments of the Archipelago of San Andres, Providence and Santa Catalina (n = 3), Magdalena (n = 16), Atlantico (n = 5), Choco (n = 3), Cauca (n = 3), Valle del Cauca (n = 4) and Nariño (n = 4). These stations were selected because they were located in sites of economic and environmental importance, such as bays, coastal lakes, swamps, low river basins and tourist beaches.

Between 0.1 and 50 were processed through membrane filtration using 0.45 µm sterile nitrocellulose filters. The filters were then transferred on a Petri dish with thiosulfate citrate bile salts sucrose (TCBS) (OxoidTM) to select for all *Vibrio* strains, as well as CHROMagarTM *Vibrio* to select for *V. cholerae*/*V. vulnificus*, *V. parahaemolyticus* and *V. alginolyticus*. The cultures were incubated at 37 ± 0.5 °C for 18 ± 4 h, then, putative *Vibrio* spp. colony-forming units (CFU) were counted. The morphotypes were replicated in new TCBS and CHROMagarTM *Vibrio* media. The isolated colonies were then transferred on a nutritive agar with 1% sodium chloride and incubated at 37 ± 0.5 °C for 18 ± 4 h. The isolates were identified by phenotypic and PCR methods and 10% of these were selected for whole genome sequencing [43].

The second program was the Intensified Cholera Surveillance program coordinated by the National Institute of Health of Colombia between 2010 and 2019, from which 14 environmental and 18 clinical samples were obtained. Sample processing was done by the Microbiology Group of National Institute of Health, following the standard protocols of the National Institute of Health [44]. The isolates were recovered by inoculation in alkaline peptone water at 8.4 pH and 37 °C for 6–8 h according to Bergey’s manual of systematic bacteriology [45]. Next, the cultures were transferred to a selective TCBS agar and incubated at 37 °C between 18 and 24 h, according to the *Vibrio cholerae* surveillance guide of the Microbiology group of the National Institute of Health [46]. The isolates were identified by phenotypic and PCR methods and 10% of these were selected for whole genome sequencing [43].

### 4.2. Whole Genome Sequencing, De Novo Assembly and Annotation

All isolates were grown in brain heart infusion (BHI) Agar overnight at 37 °C. DNA extraction was performed by boiling at 100 °C for 15 minutes from a suspension of 2–3 bacterial colonies in 100 µL of Tris HCl 0.1N to lyse the bacteria. The suspension was then centrifuged for 2 min at 12,000 rpm to precipitate the cell debris, while the supernatant was collected in a new tube for use as template and stored at 20 °C until use. DNA was extracted from each isolate using the QIAamp DNA Mini Kit (Qiagen^®,^ Hilden, Germany) following the protocol and recommendations of the manufacturer. DNA integrity and quantity were assessed on a Nanodrop UV-vis spectrophotometer (Thermo Fisher Scientific^TM^, Waltham, MA, USA) and 1% agarose gel electrophoresis visualized with ethidium bromide in a ChemiDoc XRS photodocumenter (BioRad^®^, Hercules, CA, USA). Paired-end libraries with 500 bp insert size and 250 bp read length were sequenced on an Illumina HiSeq platform, generating a raw data output of ~1 Gb per sample (~190X depth).

Prior to the genome assembly, the taxonomic assignment of each isolate was confirmed by comparing the sequenced reads against the RefSeq Bacteria database using KAIJU v1.7.3 [47]. The results were visualized with KRONA v2.7.1 [48]. We found that four isolates were contaminated with reads from *Enterobacter* spp. or *Morganella* spp.; therefore, read decontamination was done using BLAST against local databases containing all *Enterobacter* (txid: 547) or *Morganella* (txid:581) sequences available in the Nucleotide database of the National Center for Biotechnology Information (NCBI) (version August 2020). Contaminated reads were searched for using ncbi-blast v2.10.1 and non-contaminated reads were filtered and extracted using Seqtk v1.2 [49] and custom bash scripts.

Genome assembly was done according to the de novo reference-guided assembly pipeline developed and described by [50], which was modified to use SPADES v3.6.2 de novo assembler [51]. This pipeline was developed to reduce the complexity of the genomic assembly using a conspecific reference genome, without introducing bias. The pipeline spans read quality control and trimming, contig assembly and scaffolding. Briefly, raw data quality control was assessed using FastQC v0.11.4 [52] and visualized with MultiQC v1.9 [53]. Read trimming and quality filtering were done using Trimmomatic v0.36 [54] with the following parameters: HEADCROP:10 LEADING:3 TRAILING:3 SLIDINGWINDOW:4:20 MINLEN: <<100, 120 or 150>>. The minimum length was adjusted for each sample to discard the lowest number of reads possible based on overall read quality. Next, homologue regions between the reference genome and target genome were identified by read mapping using Bowtie2 v2.3.4 [55], which allowed constructing superblocks or conserved genomic regions using SAMtools v1.5 [56], BEDtools v2.25 [57] and BCFtools v1.3.1 [58]. Mapped reads were split according to the superblocks using Seqtk v1.2 and assembled separately with SPADES v3.6.2; furthermore, unmapped reads were also assembled independently. The contigs were merged into non-redundant supercontigs against the reference genome using AMOScmp v3.1.0 [59]. Finally, error correction was done with the Genome Analysis Toolkit (GATK) version [60,61] and the original reads and scaffolding was performed using MUMMER v3.23 [62] and SOAPdenovo2 v240 [63]. Each isolate was assembled using the genome reference of its species (Supplementary Table S3). Assembly quality and completeness were assessed using QUAST v4.5 [64] and BUSCO v4.0.6 [65].

Whole genome average nucleotide identity was calculated for the 60 isolates using FastANI [66]. The output was converted to a symmetrical matrix and plotted in a heatmap with hierarchical clustering in R v3.6.1 ggplot2 [67].

### 4.3. Characterization of the Most Important Species in Public Health

To determine the genetic variants (sequence types) of the most important species in public health (*V. parahaemolyticus* and *V*. *vulnificus*), MLST v2.10 [68] was used. For this, the allelic profile of housekeeping genes was obtained and a ST was signed according to the defined typing scheme for each species in PubMLST database [69]. The isolates were submitted to the database (Supplementary Tables S4 and S5).

The lineages of *V. vulnificus* were determined by a phylogenetic analysis using a database of 80 closed genomes with a defined poblational structure obtained from Roig et al. [20]. For this, core genes were aligned with Parsnp [70] and the phylogenetic tree was inferred using IQTREE v1.6.12 [71], with 1000 bootstrap replicates and the GTR + I + G model. The tree was visualized with FigTree [72]. FastANI was used to calculate the whole genome average nucleotide identity of the 80 *V. vulnificus* isolates.

### 4.4. Pangenome and Phylogenomic Analyses 

The Anvi’o workflow v2.1.0 [73] was used to build a pangenome for each species. For this, the genomes were annotated using the Clusters of Orthologues Genes (COG) database, according to the categories COG20_FUNCTION, COG20_PATHWAY and COG20_CATEGORY [74]. Three bins related to the core genes (genes present in 99–100% of the isolates), accessory genes (15–95%) and singletons (0–15%) were created and a summary of the annotation for each bin was obtained. To investigate the differences between the genomic content of accessory genomes between groups of clinical and environmental isolates within each pangenome, we created layers of additional data (e.g., source) and conducted analyses to determine significantly enriched categories (*p* value < 0.05) between these groups according to [75]. The core genes for each species were obtained from Anvi’o and used to construct a maximum likelihood (ML) phylogenetic tree using IQTREE v1.6.12 with the LG amino acid evolutionary model, I+G sites distribution and an ultrafast bootstrap of 100 replicates. The pangenome was ordered according to the phylogenetic tree obtained.

### 4.5. Identification of Virulence Factors and Antimicrobial Resistance Determinants

To identify the virulence and antibiotic resistance genes, ABRicate [76] was used with specific databases, such as NCBI AMRFinderPlus [77] and the Comprehensive Antibiotic Resistance Database (CARD) [78] for resistance determinants and the Virulence Factor Database (VFDB) [79] for virulence factors. Hits were filtered at thresholds of 80% identity and 80% coverage. As input, we used the CDS file of each genome. The predicted virulence and resistance genes were grouped according to the identity percentage and visualized with heatmap (R ggplot). This allowed us to observe the main virulence genes shared between species.

ICEfinder [36] was used to determine the presence of putative integrative and conjugative elements (ICE) and integrative and mobile elements (IME) in the assembled genomes. The association of these elements with virulence and resistance determinants was analyzed by extracting the predicted ICE or IME sequence and searching for virulence and resistance genes using VFDB (e-value = 0.01) and CARD.

## Figures and Tables

**Table 1:** Functional enrichment analyses of the accessory genome of clinical and environmental isolates of *Vibrio parahaemolyticus*.

COG20_Category	Enrichment_Score	Unadjusted_*p*_Value	Adjusted_*q*_Value	Associated_Isolate_Sources
**Cell motility|Intracellular trafficking, secretion and vesicular transport**	17	3.74 × 10^−5^	3.03 × 10^−3^	clinical
**Cell cycle control, cell division, chromosome partitioning|Cell motility**	13.388	2.53 × 10^−4^	3.16 × 10^−3^	environmental
**Amino acid transport and metabolism|Nucleotide transport and metabolism**	13.246	2.73 × 10^−4^	3.16 × 10^−3^	clinical
**Cell cycle control, cell division, chromosome partitioning|Coenzyme transport and metabolism**	13.246	2.73 × 10^−4^	3.16 × 10^−3^	clinical
**Nucleotide transport and metabolism|Signal transduction mechanisms**	13.246	2.73 × 10^−4^	3.16 × 10^−3^	clinical
**Signal transduction mechanisms|Signal transduction mechanisms**	13.246	2.73 × 10^−4^	3.16 × 10^−3^	clinical
**Carbohydrate transport and metabolism|Transcription**	13.246	2.73 × 10^−4^	3.16 × 10^−3^	environmental
**Cell cycle control, cell division, chromosome partitioning**	10.578	1.14 × 10^−3^	1.03 × 10^−2^	clinical
**Signal transduction mechanisms|Intracellular trafficking, secretion and vesicular transport**	10.578	1.14 × 10^−3^	1.03 × 10^−2^	clinical
**Intracellular trafficking, secretion and vesicular transport|General function prediction only|Transcription**	9.745	1.80 × 10^−3^	1.03 × 10^−2^	clinical
**Nucleotide transport and metabolism**	9.745	1.80 × 10^−3^	1.03 × 10^−2^	environmental

**Table 2:** Integrative elements (ICE/IME) predicted for 60 *Vibrio* spp isolates. The type “putative IME” contain predicted Integrase and Relaxase and “putative ICE with T4SS” corresponds to elements with predicted Relaxase, T4CP, Integrase and T4SS components.

Species	Isolate	Type	GC (%)	Size (kbp)	Predicted Virulence Genes (Min e-Value 0.01)
***V. parahaemolyticus***	PV1	Putative ICE	44.45	195	VP1611(MAM7)*, mshE, mshH, mshA, mshL, mshG, mshJ, tcpI*
***V. parahaemolyticus***	PV173	Putative IME	39.38	82	*vcrD2, vscC2, vopL, vscN2, vopB2, vscU2, VPA1351, vopA/vopP, VPA1353, vopZ, vopD2, vopT, vscR2, vscT2, VPA1350, vscJ2, VPA1352, VPA1331, VPA1331, VPA1363, VPA1337, VPA1340, vscS2, vscQ2, acfD, tdh, flhA, yscV/lcrD, flhA, vcrD, yscN, mxiA, flhA*
***V. parahaemolyticus***	PV278	Putative IME	40.6	35	
***V. fluvialis***	PV3	Putative ICE with T4SS	52	166	*IlpA, motB, motA*
***V. fluvialis***	PV4	Putative IME	46.86	74	
***V. fluvialis***	PV9	Putative IME	50.94	40	
***V. fluvialis***	PV50	Putative ICE with T4SS	48.48	233	*tcpI*
***V. fluvialis***	PV50	Putative IME	45.8	77	
***V. fluvialis***	PV59	Putative ICE with T4SS	47.8	215	*cqsA*
***V. fluvialis***	PV59	Putative ICE with T4SS	48.85	231	
***V. fluvialis***	PV60	Putative ICE with T4SS	48.4	296	*flaC,flaB,flgA,flgJ, flaD, flgT, flgP, flgI, flgL,flgC,flgO,flgF,flgB,cheR,cheV,flgK,flgD,flaA,flgH,flgG,tcpI,flgN*
***V. fluvialis***	PV76	Putative ICE with T4SS	47.74	226	*cqsA*
***V. fluvialis***	PV92	Putative IME with T4SS	47.99	183	*cqsA*
***V. fluvialis***	PV92	Putative IME	50.62	48	
***V. fluvialis***	PV105	Putative ICE with T4SS	49.15	165	*flgD*
***V. fluvialis***	PV131	Putative IME	47.34	52	
***V. alginolyticus***	PV126	Putative IME	41.8	75	
***V. alginolyticus***	PV242	Putative IME	45.81	15	
***V. vulnificus***	PV35	Putative IME	43.19	79	
***V. vulnificus***	PV159	Putative IME	40.53	15	
***V. vulnificus***	PV194	Putative IME	49.13	62	
***V. vulnificus***	PV207	Putative IME	40.18	57	
***V. furnissii***	PV10	Putative ICE with T4SS	53.14	173	
***V. furnissii***	PV88	Putative IME	45.96	80	
***V. furnissii***	PV169_L	Putative ICE with T4SS	51.94	388	

## Data Availability

The genomic data of the *Vibrio* isolates were deposited under BioProject ID PRJNA754786 with BioSample accessions SAMN20802061, SAMN20931810, SAMN20804964 - SAMN20804979, SAMN20805029 - SAMN20805070.

## Supplementary Materials

The following are available online at https://www.mdpi.com/article/10.3390/pathogens10121605/s1, Supplementary Table S1. Characteristics of the obtained assemblies of the six *Vibrio* species analyzed in this study. The characteristics represent statistics and metrics resulted from the quality control, preprocessing, assembly and annotation processes, Supplementary Table S2: Sample information of the *Vibrio* spp. isolates analyzed in this study, Supplementary Table S3: Reference *Vibrio* spp. genomes used for reference-guided de novo genome assemblies, Supplementary Table S4: MLST results of *Vibrio parahaemolyticus*, Supplementary Table S5: MLST results of *Vibrio vulnificus*, Figure S1: Maximum likelihood tree of 80 *V. vulnificus* isolates. BS are based on 1000 replicates. The nine isolates of the present study are marke with orange, Figure S2: Whole-genome Average Nucleotide Identity of *V. vulnificus* isolates.
