# Supplementary material for: Comparative Genomics of Clinical and Environmental Isolates of Vibrio spp. of Colombia: Implications of Traits Associated with Virulence and Resistance
Source: Pathogens. 2021 Dec 10;10(12):1605. doi: 10.3390/pathogens10121605 (PMC8706872; doi:10.3390/pathogens10121605)
Supplement: Supplementary file 1 [file pathogens-10-01605-s001.zip › FigureS1,S2.pdf]

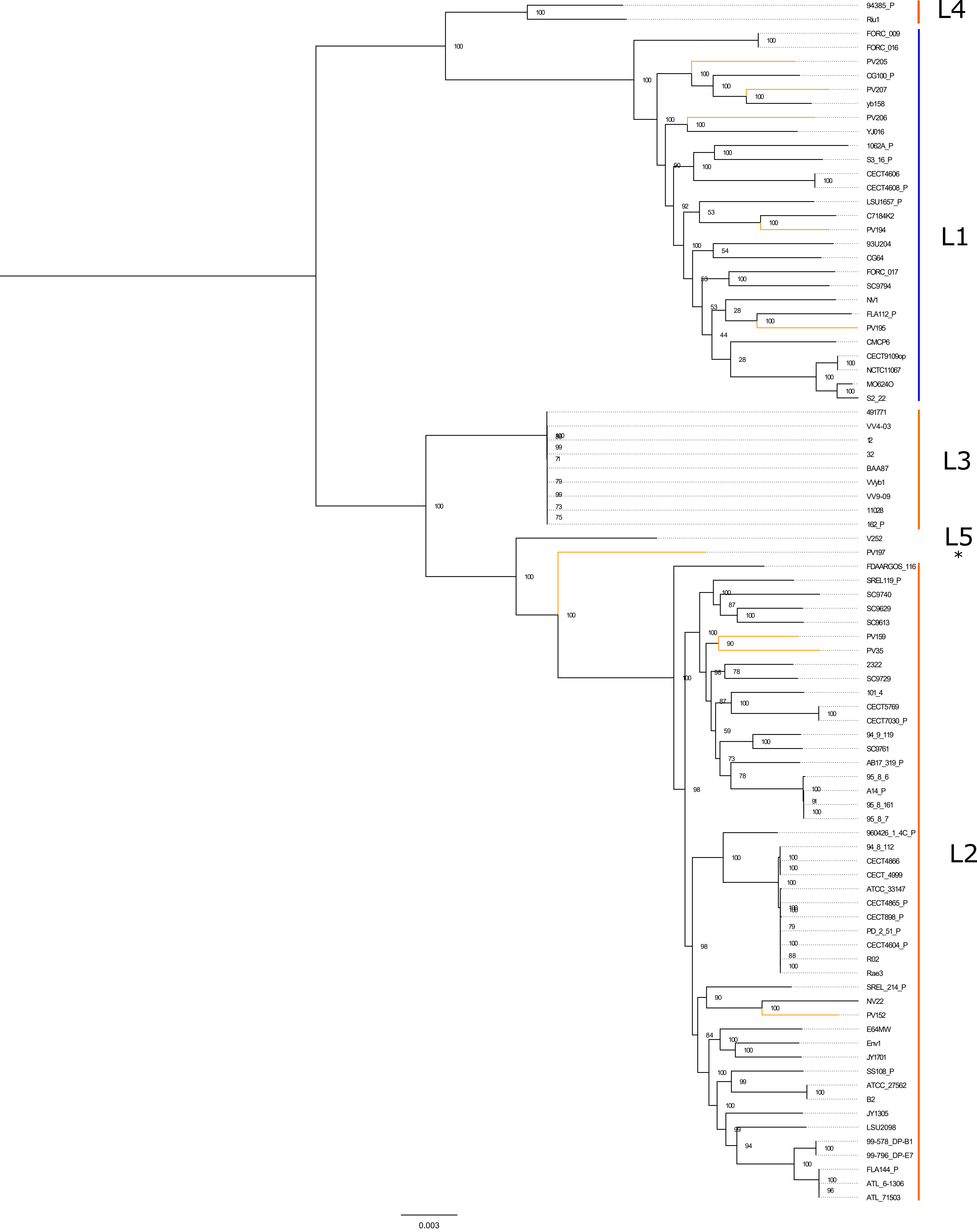

Figure S1. Maximum likelihood tree of 80 *V.vulnificus* isolates. BS are based on 1000 replicates. The nine isolates of the present study are marke with orange.

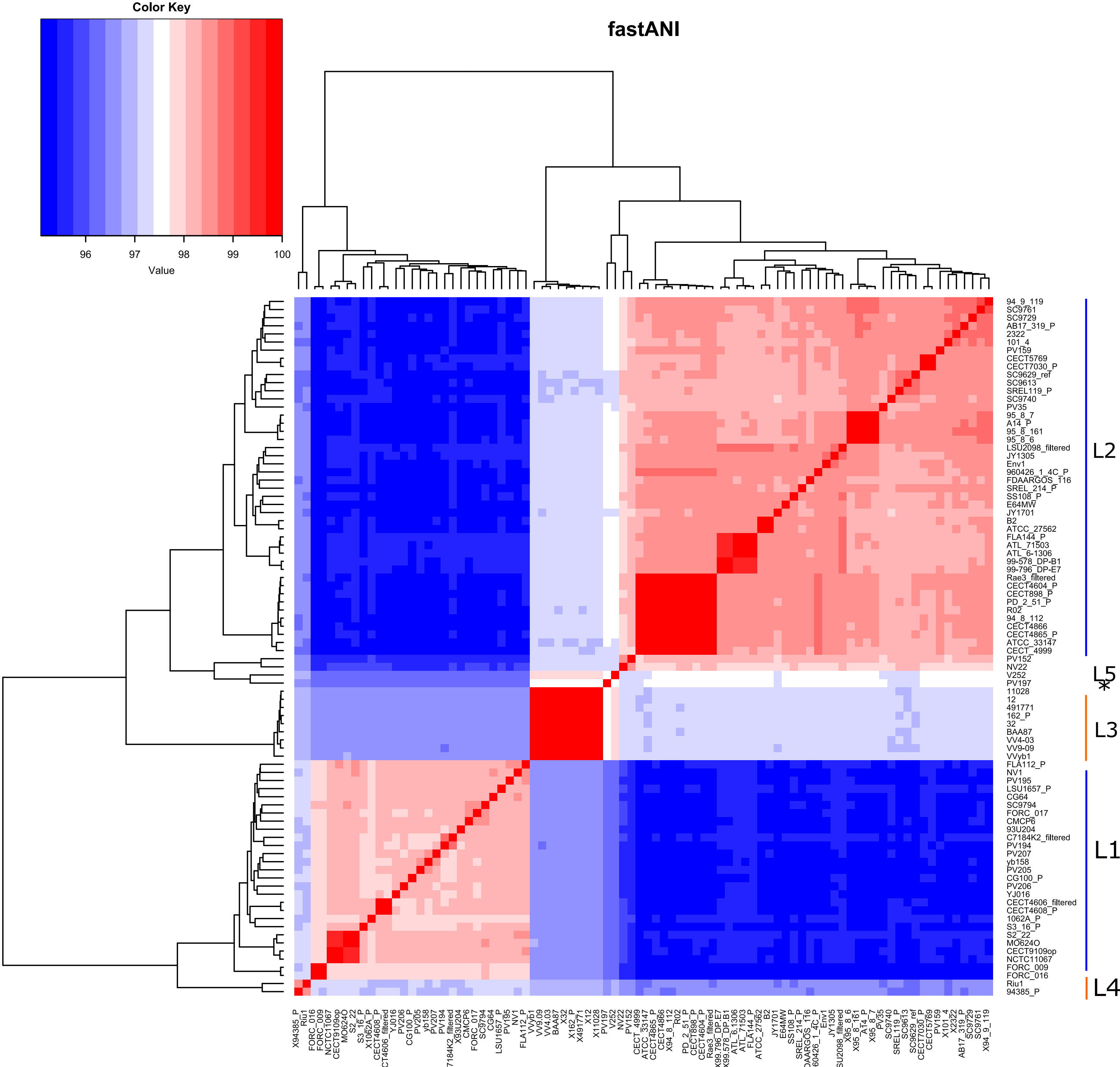

Figure S2. Whole-genome Average Nucleotide Identity of *V. vulnificus* isolates
