# Supplementary material for: Comparative Genomics of Clinical and Environmental Isolates of Vibrio spp. of Colombia: Implications of Traits Associated with Virulence and Resistance
Source: Pathogens. 2021 Dec 10;10(12):1605. doi: 10.3390/pathogens10121605 (PMC8706872; doi:10.3390/pathogens10121605)
Supplement: Supplementary file 1 [file pathogens-10-01605-s001.zip › TableS1,S2,S3,S4,S5.pdf]

Supplementary Table S1. Characteristics of the obtained assemblies of the six *Vibrio* species analyzed in this study. The characteristics represent statistics and metrics resulted from the quality control, preprocessing, assembly and annotation processes.

***Vibrio* species**	**Isolate ID**	**Assembly features**					
		**Total lenght (bp)**	**Scaffolds**	**GC content (%)**	**N50 (bp)**	**Reference genome coverage (%)**	**CDS**
V. parahaemolyticus	PV1	5128845	48	45.23	401170	91.99	4615
V. parahaemolyticus	PV11	5036579	82	45.37	164783	90.22	4569
V. parahaemolyticus	PV17	5002236	67	45.44	359608	88.79	4515
V. parahaemolyticus	PV32	5051964	82	45.34	199318	89.26	4594
V. parahaemolyticus	PV53	5133150	54	45.21	427167	97.47	4595
V. parahaemolyticus	PV85	5146837	48	45.3	426983	97.54	4635
V. parahaemolyticus	PV109	5276965	394	45.49	169396	87.51	5009
V. parahaemolyticus	PV112	5495767	270	45.47	171457	88.91	5041
V. parahaemolyticus	PV156	4997431	90	45.33	172955	87.41	4562
V. parahaemolyticus	PV161	5055780	127	45.31	138236	87.75	4638
V. parahaemolyticus	PV170	5266539	310	45.48	426973	97.47	4881
V. parahaemolyticus	PV173	5406329	537	45.63	384835	97.4	4982
V. parahaemolyticus	PV213	5247732	127	45.24	167072	89.5	4799
V. parahaemolyticus	PV221	5053872	97	45.3	162813	87.83	4626
V. parahaemolyticus	PV235	5059799	133	45.43	209984	88.61	4638
V. parahaemolyticus	PV278	5103966	45	45.25	564561	97.4	4573
V. parahaemolyticus	PV280	5144753	42	45.31	442827	97.52	4642
V. fluvialis	PV3	4786892	91	50.03	193060	89.57	4480
V. fluvialis	PV4	4648859	63	50.07	248249	89.28	4323
V. fluvialis	PV5	4648576	60	50.1	202241	89.04	4352

V. fluvialis	PV7	4715369	59	50.02	185239	89.25	4403
V. fluvialis	PV8	4649633	69	50.1	201884	89.05	4345
V. fluvialis	PV9	4633335	63	50.15	174774	89.59	4280
V. fluvialis	PV47	4758672	54	50.02	214306	88.22	4466
V. fluvialis	PV50	4674347	62	50	189585	91.68	4334
V. fluvialis	PV59	4723217	47	50.1	410476	89.67	4418
V. fluvialis	PV60	4808719	61	49.95	181182	91.4	4490
V. fluvialis	PV75	4827992	90	49.87	145719	90.23	4506
V. fluvialis	PV76	5029615	238	49.89	117824	90.99	4843
V. fluvialis	PV92	4674008	57	50.14	251845	89.96	4338
V. fluvialis	PV101	4679663	77	50.11	209951	89.31	4417
V. fluvialis	PV105	4739424	168	50.15	268904	89.8	4560
V. fluvialis	PV131	4841678	111	50.04	146095	91.14	4503
V. alginolyticus	PV82	5077839	85	44.67	206900	91.4	4564
V. alginolyticus	PV116	5246251	241	44.85	303610	94.19	4877
V. alginolyticus	PV118	5129110	81	44.61	210035	94.25	4637
V. alginolyticus	PV126	5128107	74	44.6	212144	94.22	4617
V. alginolyticus	PV153	5096286	146	44.64	177533	90.51	4672
V. alginolyticus	PV178	5146549	80	44.57	172036	91.04	4656
V. alginolyticus	PV242	5298417	103	44.62	116907	88.53	4785
V. alginolyticus	PV255	5108897	81	44.6	165308	92.19	4582
V. vulnificus	PV35	5002584	203	46.74	109370	83.59	4459
V. vulnificus	PV152	4768546	225	46.61	75474	80.95	4304
V. vulnificus	PV159	4821910	169	46.75	99144	83.74	4279
V. vulnificus	PV194	5107101	301	46.79	84019	59.31	4547
V. vulnificus	PV195	4897715	146	46.89	105901	54.7	4397
V. vulnificus	PV197	5103500	330	46.8	65195	80.73	4537
V. vulnificus	PV205	4883385	218	46.99	106918	55.96	4369

V. vulnificus	PV206	5062232	217	46.84	89701	55.22	4570
V. vulnificus	PV207	4974440	193	46.91	91250	55.5	4477
V. furnissii	PV10	5014480	75	50.4	172263	90.9	4646
V. furnissii	PV57	4930994	74	50.84	163279	90.61	4543
V. furnissii	PV88	4816633	69	50.82	167877	89.93	4442
V. furnissii	PV169_L	4881141	79	50.63	223193	90.58	4493
V. furnissii	PV169_S	4881141	79	50.63	223193	90.58	4493
V. furnissii	PV231	5226034	105	50.23	184803	90.57	4954
V. diabolicus	PV89	5052288	56	44.84	226153	90.58	4568
V. diabolicus	PV164	5159768	290	45.07	217868	89.63	4909
V. diabolicus	PV269	5408210	491	44.91	100179	89.13	4992
V. diabolicus	PV270	5135936	104	44.68	141052	88.98	4703

Supplementary Table S2. Sample information of the *Vibrio* spp. isolates analyzed in this study.

Vibrio species	Isolate ID	Sample features						NCBI Biosample accession number
		Collection date	Location	Sample type	Source	Virulence genes	Reference	
V. parahaemolyticus	PV1	27/04/2011	Santa Marta	Clinical	Stool	*tdh + trh* -	LSPD	SAMN20804966
V. parahaemolyticus	PV11	05/09/2012	Magdalena	Environmental	Continental Water	*tdh - trh -*	LSPD	SAMN20805030
V. parahaemolyticus	PV17	07/09/2018	Choco	Environmental	Continental Water	*tdh - trh -*	INVEMAR	SAMN20805036
V. parahaemolyticus	PV32	17/09/2018	ND	Environmental	Continental Water	*tdh - trh -*	INVEMAR	SAMN20805037
V. parahaemolyticus	PV53	30/07/2018	Cordoba	Clinical	Stool	*tdh + trh* -	LSPD	SAMN20804969
V. parahaemolyticus	PV85	16/10/2017	Cordoba	Clinical	Stool	*tdh + trh* -	LSPD	SAMN20804970
V.parahaemolyticus	PV109	07/09/2018	Choco	Environmental	Marine water	*tdh - trh -*	INVEMAR	SAMN20802061
V. parahaemolyticus	PV112	07/09/2018	Choco	Environmental	Marine water	*tdh - trh -*	INVEMAR	SAMN20805029
V. parahaemolyticus	PV156	22/04/2019	Atlantico	Environmental	Estuarine water	*tdh - trh -*	INVEMAR	SAMN20805031
V. parahaemolyticus	PV161	22/04/2019	Atlantico	Environmental	Continental Water	*tdh - trh -*	INVEMAR	SAMN20805035
V. parahaemolyticus	PV170	06/05/2019	Cordoba	Clinical	Stool	*tdh + trh* -	LSPD	SAMN20804964
V. parahaemolyticus	PV173	17/07/2019	Cordoba	Clinical	ND	*tdh + trh* -	LSPD	SAMN20804965
V. parahaemolyticus	PV213	26/06/2019	Valle del Cauca	Environmental	Estuarine water	*tdh - trh -*	INVEMAR	SAMN20805032
V. parahaemolyticus	PV221	22/04/2019	Atlantico	Environmental	Continental Water	*tdh - trh -*	INVEMAR	SAMN20805033
V. parahaemolyticus	PV235	02/05/2019	Nariño	Environmental	Continental Water	*tdh - trh -*	INVEMAR	SAMN20805034
V. parahaemolyticus	PV278	30/07/2019	Bogota D.C.	Clinical	Stool	*tdh + trh* -	LSPD	SAMN20804967
V. parahaemolyticus	PV280	24/07/2019	Cordoba	Clinical	Stool	*tdh + trh* -	LSPD	SAMN20804968
V. fluvialis	PV3	08/12/2014	Atlantico	Environmental	Continental water	*vfh +*	LSPD	SAMN20805038

V. fluvialis	PV4	26/04/2011	San Andres	Environmental	Water	*vfh +*	LSPD	SAMN20805040
V. fluvialis	PV5	17/02/2011	Cauca	Environmental	Continental water	*vfh +*	LSPD	SAMN20805042
V. fluvialis	PV7	09/05/2011	San Andres	Environmental	Continental water	*vfh +*	LSPD	SAMN20805046
V. fluvialis	PV8	02/02/2011	ND	ND	ND	*vfh +*	LSPD	SAMN20805047
V. fluvialis	PV9	14/04/2011	Cauca	Clinical	Stool	*vfh +*	LSPD	SAMN20804975
V. fluvialis	PV47	14/03/2018	Magdalena	Environmental	Marine water	*vfh +*	LSPD	SAMN20805039
V. fluvialis	PV50	22/05/2018	La Guajira	Clinical	Stool	*vfh +*	LSPD	SAMN20804974
V. fluvialis	PV59	18/11/2018	Magdalena	Environmental	Untreated water	*vfh +*	LSPD	SAMN20805041
V. fluvialis	PV60	18/11/2018	Magdalena	Environmental	Untreated water	*vfh +*	LSPD	SAMN20805043
V. fluvialis	PV75	29/06/2017	San Andres	Environmental	Water	*vfh +*	LSPD	SAMN20805044
V. fluvialis	PV76	05/07/2017	Magdalena	Environmental	Water	*vfh +*	LSPD	SAMN20805045
V. fluvialis	PV92	09/05/2017	Magdalena	Environmental	Fresh water	*vfh +*	LSPD	SAMN20805048
V. fluvialis	PV101	11/02/2016	Nariño	Clinical	Stool	*vfh +*	LSPD	SAMN20804971
V. fluvialis	PV105	31/10/2016	San Andres	Clinical	Stool	*vfh +*	LSPD	SAMN20804972
V. fluvialis	PV131	03/04/2019	Amazonas	Clinical	Stool	*vfh +*	LSPD	SAMN20804973
V. alginolyticus	PV82	10/10/2017	Magdalena	Environmental	Marine water	*tdh -*	LSPD	SAMN20805049
V. alginolyticus	PV116	07/09/2018	Choco	Environmental	Continental water	*tdh -*	INVEMAR	SAMN20805050
V. alginolyticus	PV118	27/08/2018	Magdalena	Environmental	Marine water	*tdh -*	INVEMAR	SAMN20805051
V. alginolyticus	PV126	28/08/2018	Magdalena	Environmental	Marine water	*tdh -*	INVEMAR	SAMN20805052
V. alginolyticus	PV153	23/04/2019	Atlantico	Environmental	Continental water	*tdh -*	INVEMAR	SAMN20805053
V. alginolyticus	PV178	22/04/2019	Atlantico	Environmental	Marine water	*tdh -*	INVEMAR	SAMN20805054
V. alginolyticus	PV242	02/05/2019	Nariño	Environmental	Water	*tdh -*	INVEMAR	SAMN20805055
V. alginolyticus	PV255	02/05/2019	Nariño	Environmental	Water	*tdh -*	INVEMAR	SAMN20805056
V. vulnificus	PV35	12/10/2018	Santander	Clinical	Wound	*vcgE +*	LSPD	SAMN20804976
V. vulnificus	PV152	22/04/2019	Atlantico	Environmental	Estuarine water	*vcgC +*	INVEMAR	SAMN20805057

V. vulnificus	PV159	22/04/2019	Atlantico	Environmental	Water	*vcgE +*	INVEMAR	SAMN20805058
V. vulnificus	PV194	26/06/2019	Valle del Cauca	Environmental	Water	*vcgC +*	INVEMAR	SAMN20805059
V. vulnificus	PV195	26/06/2019	Valle del Cauca	Environmental	Water	*vcgC +*	INVEMAR	SAMN20805060
V. vulnificus	PV197	26/06/2019	Valle del Cauca	Environmental	Water	*vcgC +*	INVEMAR	SAMN20805064
V. vulnificus	PV205	26/06/2019	Valle del Cauca	Environmental	Marine water	*vcgC +*	INVEMAR	SAMN20805061
V. vulnificus	PV206	26/06/2019	Valle del Cauca	Environmental	Marine water	*vcgC +*	INVEMAR	SAMN20805062
V. vulnificus	PV207	26/06/2019	Valle del Cauca	Environmental	Marine water	*vcgC +*	INVEMAR	SAMN20805063
V. furnissii	PV10	11/04/2011	La Guajira	Environmental	Water	*vfh +*	LSPD	SAMN20805065
V. furnissii	PV57	22/09/2018	Norte de Santander	Clinical	Stool	*vfh +*	LSPD	SAMN20804978
V. furnissii	PV88	09/11/2017	Choco	Clinical	Stool	*vfh +*	LSPD	SAMN20804979
V. furnissii	PV169_S	07/06/2019	Nariño	Clinical	Stool	*vfh +*	LSPD	SAMN20931810
V. furnissii	PV169_L	07/06/2019	Nariño	Clinical	Stool	*vfh +*	LSPD	SAMN20931810
V. furnissii	PV231	02/05/2019	Nariño	Environmental	Water	*vfh +*	INVEMAR	SAMN20805066
V. diabolicus	PV164	22/04/2019	Atlantico	Environmental	Water	*tdh -*	INVEMAR	SAMN20805067
V. diabolicus	PV269	28/05/2019	Magdalena	Environmental	Marine water	*tdh -*	INVEMAR	SAMN20805068
V. diabolicus	PV270	28/05/2019	Magdalena	Environmental	Marine water	*tdh -*	INVEMAR	SAMN20805069
V. diabolicus	PV89	12/12/2017	Magdalena	Environmental	Marine water	*tdh -*	LSPD	SAMN20805070

Reference: INVEMAR: José Benito Vives de Andréis Marine and Coastal Research Institute. LSPD: Laboratorio de Salud Pública - Secretaria Distrital de Salud de Bogota. The data were extracted from: Grupo de Microbiología, Instituto Nacional de Salud de Colombia, base de datos proyecto “*Vibrio* spp en reservorios de agua en Colombia, como agentes potenciales de cólera y vibriosis”. 2020. Datos no publicados

Supplementary Table S3. Reference *Vibrio* spp. genomes used for reference-guided de novo genome assemblies.

**Vibrio species**	**RefSeq accessions for chromosomes 1, 2 and plasmid, if present**	**GenBank assembly accession**
Vibrio parahaemolyticus	NC_004603.1	GCA_000196095.1
	NC_004605.1	
Vibrio alginolyticus	NC_022349.1	GCF_000354175.2
	NC_022359.1	
Vibrio fluvialis	NZ_CP014034.2	GCF_001558415.2
	NZ_CP014035.2	
Vibrio vulnificus	NZ_CP014636.1	GCF_002215135.1
	NZ_CP014637.1	
	NZ_CP014638.1 (plasmid)	
Vibrio diabolicus	NZ_CP042451.1	GCF_011801455.1
	NZ_CP042452.1	
Vibrio furnissii	NZ_CP040990.1	GCF_006364355.1
	NZ_CP040991.1	
	NZ_CP040991.1 (plasmid)	

Supplementary Table S4. MLST results of *Vibrio parahaemolyticus*.

Isolate	ST	*dnaE*	*gyrB*	*recA*	*dtdS*	*pntA*	*pyrC*	*tnaA*	source	year	pubmlst_id
PV109	2486	155	475	242	407	206	11	24	environment	2018	3578
PV11	2487	12	180	81	53	21	11	17	environment	2012	3579
PV156	2508	406	90	410	353	26	295	9	environment	2019	3617
PV161	2489	31	124	411	76	134	194	24	environment	2019	3625
PV170	3	3	4	19	4	29	4	22	clinical	2019	3618
PV173	3	3	4	19	4	29	4	22	clinical	2019	3619
PV17	-	10	~510	335	~330	~50	18	~33	environment		UT
PV1	120	60	108	86	98	18	45	51	clinical	2011	3620
PV213	2488	289	563	3	13	230	54	24	environment	2019	3580
PV221	2489	31	124	411	76	134	194	24	environment	2019	3581
PV235	2490	148	562	31	510	23	461	17	environment	2019	3582
PV278	3	3	4	19	4	29	4	22	clinical	2019	3621
PV280	3	3	4	19	4	29	4	22	clinical	2019	3622
PV32	2491	416	104	67	251	219	206	24	environment	2018	3583
PV53	3	3	4	19	4	29	4	22	clinical	2018	3623
PV85	3	3	4	19	4	29	4	22	clinical	2017	3624
PV112	-	10	~510	335	~330	~50	18	~33	environment		UT

Supplementary Table S5. MLST results of *Vibrio vulnificus*.

Isolate	ST	*glp*	*gyrB*	*mdh*	*metG*	*purM*	*dtdS*	*lysA*	*pntA*	*pyrC*	*tnaA*	source	year	pubmlst_id
PV152	571	104	42	95	103	23	193	101	25	106	18	enviroment	2019	776
PV159	572	138	14	11	9	8	194	184	113	11	22	enviroment	2019	777
PV194	598	5	30	8	1	1	8	9	1	10	7	enviroment	2019	806
PV195	599	3	112	3	25	91	26	2	1	29	7	enviroment	2019	804
PV197	573	139	113	6	105	4	195	185	114	142	127	enviroment	2019	778
PV205	600	35	1	121	1	26	39	35	39	141	13	enviroment	2019	805
PV206	601	100	3	120	10	53	143	141	10	140	91	enviroment	2019	806
PV207	335	22	3	22	19	20	29	2	36	3	7	enviroment	2019	807
PV35	574	80	77	2	104	8	22	12	5	12	45	clinical	2018	779
